# Spatio-Temporal Variation of the Bacterial Communities along a Salinity Gradient within a Thalassohaline Environment (Saline di Tarquinia Salterns, Italy)

**DOI:** 10.3390/molecules26051338

**Published:** 2021-03-02

**Authors:** Susanna Gorrasi, Andrea Franzetti, Roberto Ambrosini, Francesca Pittino, Marcella Pasqualetti, Massimiliano Fenice

**Affiliations:** 1 Dipartimento di Ecologia e Biologia, Università degli Studi della Tuscia, Largo Università snc, 01100 Viterbo, Italy; gorrasi@unitus.it (S.G.); mpasqual@unitus.it (M.P.); 2 Dipartimento di Scienze dell’Ambiente e della Terra, Università di Milano-Bicocca, Piazza della Scienza 1, 20126 Milano, Italy; andrea.franzetti@unimib.it (A.F.); f.pittino@campus.unimib.it (F.P.); 3 Dipartimento di Scienze e Politiche Ambientali, Università degli Studi di Milano, Via Celoria 2, 20133 Milano, Italy; roberto.ambrosini@unimi.it; 4 Laboratoro di Ecologia dei Funghi Marini CONISMA, Università degli Studi della Tuscia, Largo Università snc, 01100 Viterbo, Italy; 5 Laboratorio di Microbiologia Marina Applicata, CONISMA, Università degli Studi della Tuscia, Largo Università snc, 01100 Viterbo, Italy

**Keywords:** bacterial communities, community composition, community structure, hypersaline environment, marine salterns, Saline di Tarquinia, salinity gradient, metabarcoding analysis, Illumina MiSeq

## Abstract

The “Saline di Tarquinia” salterns have been scarcely investigated regarding their microbiological aspects. This work studied the structure and composition of their bacterial communities along the salinity gradient (from the nearby sea through different ponds). The communities showed increasing simplification of pond bacterial diversity along the gradient (particularly if compared to those of the sea). Among the 38 assigned phyla, the most represented were *Proteobacteria*, *Actinobacteria* and *Bacteroidetes*. Differently to other marine salterns, where at the highest salinities *Bacteroidetes* dominated, preponderance of *Proteobacteria* was observed. At the genus level the most abundant taxa were *Pontimonas*, *Marivita*, *Spiribacter*, *Bordetella*, *GpVII* and *Lentibacter*. The α-diversity analysis showed that the communities were highly uneven, and the Canonical Correspondence Analysis indicated that they were structured by various factors (sampling site, sampling year, salinity, and sampling month). Moreover, the taxa abundance variation in relation to these significant parameters were investigated by Generalized Linear Models. This work represents the first investigation of a marine saltern, carried out by a metabarcoding approach, which permitted a broad vision of the bacterial diversity, covering both a wide temporal span (two years with monthly sampling) and the entire salinity gradient (from the nearby sea up to the crystallisation ponds).

## 1. Introduction

Marine salterns (MS) are transition areas subject to very intense daily and seasonal variations of environmental parameters, particularly water temperature and salinity. Few other extreme environments undergo fluctuations of water availability and temperature as extreme as MS [1,2,3], which expose organisms to high environmental stress. Thus MS, besides being excellent models to study microbial diversity and ecology at different salt concentrations, are also suitable models to investigate phenomena such as scarce water availability and desiccation linked to currently ongoing global change [3,4].

Organisms of MS include both marine and more specialized species adapted to the high salinity [5,6,7,8]. The species richness of multicellular organisms decreases when salinity increases; at the highest salinities, animals and plants disappear and life is represented exclusively by Archaea, Bacteria and unicellular Eukaryotes [9]. However, the microorganism communities in the different ponds are due to both the active growth of resident taxa (specifically adapted to the pond environmental conditions) and to external inputs from neighbouring ponds, atmospheric events and bird droppings.

MS usually present a series of shallow ponds sequentially connected in order to establish an increasing salinity gradient; seawater is transferred through low salinity ponds (concentrators), to ponds where salt precipitation occurs (crystallisers) [10]. MS are therefore highly heterogeneous environments inhabited by complex biological communities distributed and differentiated along the salinity gradient, which also represents an environmental barrier difficult to cross both for macro- and micro-organisms [11,12]. In these environments, salt concentration clearly exerts a high selective pressure on organisms and is therefore the main environmental factor shaping the structure and the composition of the microbial communities [13]. Overall, prokaryotes, which dominate communities at the highest salinity levels, represent useful models to study the adaptive strategies used to cope with the above-mentioned severe life conditions and the relative population dynamics [14,15,16].

Various studies have been carried out by culture-independent methods to assess the prokaryotic diversity of MS. However, the majority were based on low-resolution methods (i.e., 16S rDNA clone libraries or Denaturing Gradient Gel Electrophoresis, DGGE) [17,18,19,20,21,22,23,24,25,26,27], which returned a limited vision of the diversity, affecting also community structure analysis. In addition, the majority of the investigations are based on one-time sampling [17,18,19,21,23,25,27,28,29,30,31,32,33,34,35], generating scant information on the prokaryotic taxa fluctuations over time.

Moreover, general prokaryotic community pattern and distribution along the salinity gradient within the MS environments have often been described [4,17,22,25,28,31,36,37]. Besides, it has been shown that MS of different geographic areas revealed only partially similar patterns and distribution of prokaryotes [18,20,21,23,26,27,32,33,34,35,38,39]. Thus, tracing and identifying common and different features in the prokaryotic assemblages of the worldwide MS is of valuable biological and ecological interest.

The present work specifically investigated the halophilic bacterial diversity of the Saline di Tarquinia marine salterns and its variation, over two years, through a metabarcoding approach.

The “Saline di Tarquinia” salterns (ST) cover an area of about 135 ha and are located at ca. 80 km NW of Rome, along a low sandy coast of the North Tyrrhenian Sea, from which they are separated by an array of low dunes. The salinity gradient is developed along a series of ca. 100 shallow interconnected ponds [16]. The industrial salt production in ST started in 1805 (but historical sources documented salt production already during the Etruscan and Roman ages) and ceased in 1997. In 1980 ST become a Nature Reserve. Concomitantly, anthropic activities dramatically reduced and the management and maintenance of the pond water balance changed (i.e., control of seawater inputs become irregular). Over time, the entire site structure was altered. In particular, the levees between the various ponds were eroded, allowing mixing of waters with different salinity. However, ST can still be considered an extreme environment with broad and sudden variations of environmental parameters (in particular salinity and water temperature) in the majority of the ponds [3].

ST biodiversity has been studied since 1976, and in 2007 a research laboratory was established in ST to coordinate and optimise the research activities. However, despite the intense research activity, the microbial communities of the sites were poorly investigated. Indeed, the majority of the studies regarded ST plant and animal diversity and ecology [6,40,41,42,43,44,45,46,47,48], and only a few studies regarded the presence and characterisation of phytoplanktonic organisms [8,40,49,50]. Only a preliminary investigation, carried out both by culture-dependent and culture-independent methods (DGGE), provided a first characterisation of the bacterial community found in some ponds [7]. Subsequently, a more detailed characterisation of the cultivable fraction was performed, analysing the ability of various strains to cope with temperature and salinity variations [3]. In addition, a recent survey regarding the *Vibrio* community in different ponds of the salterns was carried out [51].

The aim of this work was to provide the first in-depth characterisation (by a metabarcoding approach) of the bacterial communities of the Saline di Tarquinia salterns. The salinity variations occurring in this thalassohaline environment make ST an ideal environment to study how the bacterial communities cope with fluctuating salinities, monitoring their spatial and temporal variations. The value of this study is the broad investigation of bacterial community variations, both considering the salinity gradient and the wide time span.

## 2. Results

The work investigated the temporal (over two years) and spatial (along the salinity gradient) variation of the bacterial communities within the “Saline di Tarquinia”, a MS located in central Italy on the Tyrrhenian coast (North of Rome). Water samples were collected monthly in the period May 2012–April 2014 from the nearby sea (feeding the ST saltern system) and among the series of interconnected ponds, from three non-consecutive ponds selected in order to cover the ST salinity gradient: a low-salinity concentrator pond (P5), an intermediate-salinity concentrator pond (P24) and a crystallisation pond (P37). During the two sampling years, the ponds showed salinity fluctuations (Table S1, Supplementary Materials) with values in the ranges 20%–48‰ (P5), 38%–142‰ (P24) and 35%–336‰ (P37). However, except for some months (Table S1, Supplementary Materials) where P37 salinity were rather lower than P24 salinity, increasing salinity values were recorded from the low-salinity concentrator pond to the crystallisation pond.

### 2.1. Composition of the Bacterial Communities

A total of 2,539,445 mapping sequences was obtained from the monthly survey of the bacterial communities along the salinity gradient, over the period May 2012–April 2014. Overall, Good’s index ranged from 90.2 to 99.6, indicating a good coverage for the libraries. The clustering sequence process defined 13,942 bacterial operational taxonomic units (OTUs).

OTUs retrieved were assigned to 38 phyla, 78 classes, 148 orders, 335 families and 1052 genera. At the phylum level, unassigned OTUs ranged from 0.6% to 4.2% in the sea, from 0.3% to 3.5% in P5, from 0.3% to 13% in P24 and from 0.1% to 20.4% in P37.

**Figure 1:**
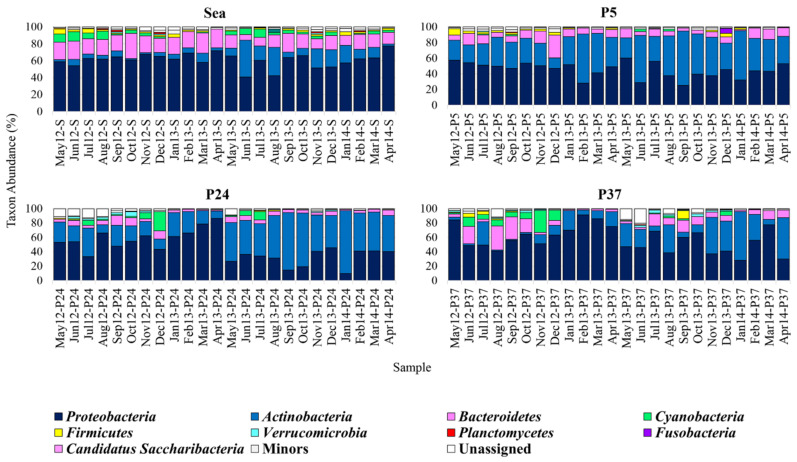
Bacterial composition of the Saline di Tarquinia (ST) samples at the Phylum-level. Distribution of the major phyla (Ra ≥ 1% in at least one sample); phyla with Ra < 1% were gathered in “Others”.

At the phylum level, the major taxa (relative abundance (Ra) ≥ 1% in at least one sample) found in ST were *Proteobacteria*, *Actinobacteria*, *Bacteroidetes*, *Cyanobacteria*, *Firmicutes*, *Verrucomicrobia*, *Planctomycetes*, *Fusobacteria*, *Candidatus Saccharibacteria* and *Deinococcus-Thermus* (Figure 1). Among them, *Proteobacteria*, *Actinobacteria* and *Bacteroidetes* were the most represented and were always recorded with a Ra ≥ 1% in all sampling sites over the two-year campaign.

In the sea samples (38%–40‰ salinity), except for June 2013, when *Actinobacteria* predominated (Ra = 43.5%), *Proteobacteria* was always the most abundant phylum (40.8–77.4%). *Bacteroidetes* most often represented the second abundant taxon (6.5–29.8%), except in the periods June-August 2013 and November 2013–January 2014, characterised by a higher abundance of *Actinobacteria*.

In P5 (20%–48‰ salinity), the bacterial communities were dominated by *Proteobacteria* and *Actinobacteria*, representing the most abundant phylum in 67% and 33% of samples, respectively. In particular, *Proteobacteria* dominated in the periods May 2012–January 2013, April–May 2013, July 2013, December 2013 and February–April 2014. This was generally followed by *Actinobacteria*; the only exception was in December 2012, where a peak of *Bacteroidetes* (Ra = 29.3%) was recorded.

In P24 (38%–142‰ salinity), the bacterial communities were dominated by *Proteobacteria* or *Actinobacteria*. *Proteobacteria* represented the most abundant phylum in the periods May–June 2012, August 2012–April 2013, and December 2013, whereas *Actinobacteria* dominated in the remaining months. In general, in months characterised by *Proteobacteria* preponderance, *Actinobacteria* was the second most abundant phylum, and vice versa. The only exception was in December 2012, when the second most abundant phylum was *Cyanobacteria* (Ra = ~27%).

Communities in P37 (35–336‰ salinity) were also dominated by *Proteobacteria* and *Actinobacteria* in 83% and 17% of samples, respectively. *Proteobacteria* was the most abundant phylum during the first sampling year (May 2012–April 2013), in July 2013 and in the periods August–September 2013 and February–March 2014. *Actinobacteria* predominated in all remaining months and mostly represented the second most abundant phylum (mainly in the second sampling year). *Bacteroidetes* represented the second most abundant phylum in summer and autumn (Jun 2012, August–October 2012, and July–October 2013). Although *Proteobacteria*, *Actinobacteria* and *Bacteroidetes* were generally the most represented phyla, a strong peak of *Cyanobacteria* (Ra = ~30%) was detected in November 2012.

In general, in all sites an increase of *Actinobacteria* was observed in the second sampling year (May 2013–April 2014), accompanied by the concomitant decrease of *Proteobacteria*, and sometimes of *Bacteroidetes* (although to a lesser extent) (Figure 1); this was particularly evident in P24.

Overall, considering all sampling sites, *Proteobacteria* abundance was higher in the sea than in the ponds (F_3,79_ = 12.607, *p*_FDR_ < 0.001; Figure S1, Supplementary Materials). However, the abundance variations of this taxon, quite limited in the sea, were rather evident in the ST ponds (they were much broader in P24 and P37).

*Proteobacteria*, with some exceptions, seemed to be more abundant at low-intermediate salinities (26%–180‰) (Figure S2, Supplementary Materials). On the contrary, abundance of *Actinobacteria* was statistically higher in the ponds than in the sea (F_3,79_ = 41.343, *p*_FDR_ < 0.001), although showing the broadest variations in P24 and P37 (Figure S1, Supplementary Materials). Abundance of *Actinobacteria* seemed to be higher also at mainly low-intermediate salinities (26%–146‰) (Figure S3, Supplementary Materials).

Compared to *Proteobacteria* and *Actinobacteria*, *Bacteroidetes* showed more limited abundance variations within the ST communities. However, its abundance was generally higher in the sea than in the ponds (F_3,79_ = 17.793, *p*_FDR_ < 0.001) (Figure S1, Supplementary Materials). In P37, *Bacteroidetes* showed the broadest abundance variations. Moreover, peaks of its abundance were recorded in some months characterised by highest salinities (Figure S4, Supplementary Materials).

With the sole exception of February 2013 and April 2014 in P24, *Cyanobacteria* was always detected (Figure 1); its abundances were lower in P5 than in the other sampling sites (F_3,79_ = 11.251, *p*_FDR_ < 0.001) (Figure S1, Supplementary Materials). In the sea, it represented a major phylum in 19 out of 24, being more abundant from late spring to early autumn (the highest abundances, Ra > 6%, were recorded in the periods May–August 2012 and May–July 2013). P5 definitely revealed the lowest occurrence of this phylum: it was usually detected as a rare taxon (Ra < 1%) excluding in July 2012, September 2012, June 2013 and August 2013, where its abundances were in the range 1.3%–2.8%. In P24 and P37 *Cyanobacteria* was recorded with some notable peaks in some months only.

In P24, except for July 2012, November–December 2012 and June–July 2013, in which abundances were greater than 6% (peak in December 2012, Ra = ~27%), *Cyanobacteria* were always rather low or a minor taxon. In P37 it was always major taxon at salinities higher than 180‰ (with Ras in the range 1.5–11.2%).

Abundance of *Firmicutes* was on average higher in the sea and P5 than in P24 and P37 (F_3,79_ = 18.651, *p*_FDR_ < 0.001) (Figure 1; Figure S1, Supplementary Materials). In the sea, it ranged from ~0.6% to ~5.8%, having an average abundance of about 2%. It represented a rare taxon only in three months (April, June and August 2013). In P5, it ranged from ~0.1% to ~8.2%, with an average abundance of about 1.8%, and it represented a rare taxon in 11 out 24 months. In P24, it ranged from ~0.1% to ~1.7%, being definitely lower than in the other sampling sites. It usually represented a rare taxon, except for May–June 2012 and July–August 2013. In P37, *Firmicutes* ranged from ~0.2% to ~11.2% and was mostly a rare taxon, being among major phyla only in the first 5 months, and in September 2013 (when a peak was detected; Ra = ~11%).

*Verrucomicrobia* was found in all samples, but often it represented a rare taxon (Figure 1); this was particularly true for the sea and P5. It was detected as a major taxon mainly in P24 and P37: in P24, during the period June–November 2012; in P37 in May 2012 and in the periods August–November 2012 and June–December 2013.

With the only exception of September 2012 in P5, *Planctomycetes* always represented a rare taxon in the ponds, whereas in the sea it represented a major taxon in August–September 2012, December 2012, November 2013 and February 2014 (Figure 1).

*Fusobacteria* and *Candidatus Saccharibacteria* always represented a rare taxon, excluding the peaks recorded in December 2013 for *Fusobacteria* in P5 (Ra = 6%) and in August 2013 for *Candidatus Saccharibacteria* in the sea (Ra = 4.2%) (Figure 1).

**Figure 2:**
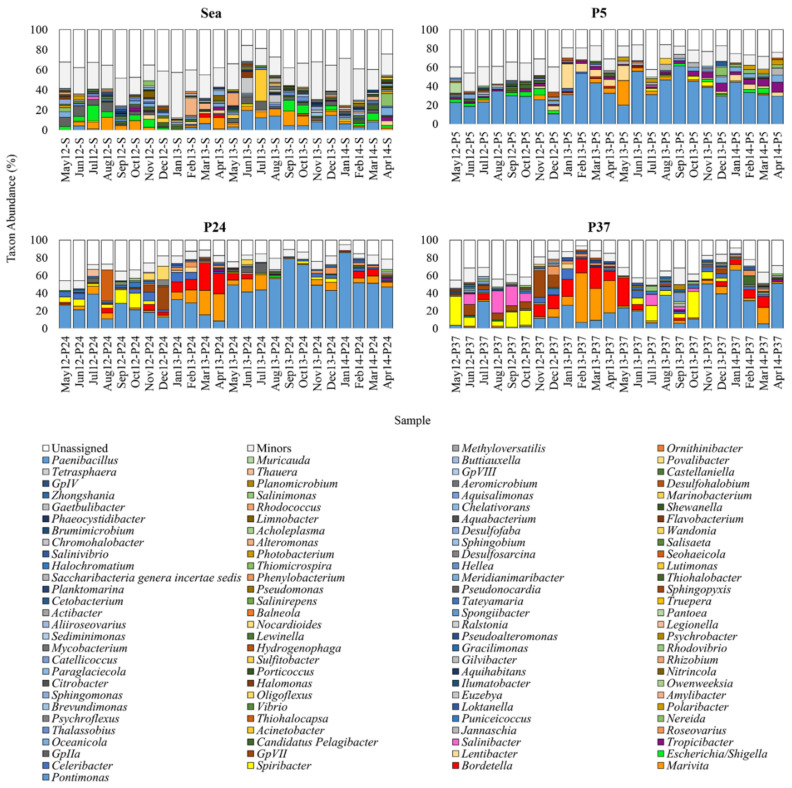
Bacterial composition of the ST samples at the Genus-level. Distribution of the major genera (Ra ≥ 1% in at least one sample); genera with Ra < 1% were gathered in “Others”.

Among the 1052 genera globally retrieved, 111 had Ra ≥ 1% in at least one sample; unassigned OTUs ranged from 15.8% to 48.5% in the sea, from 16% to 45.9% in P5, from 5.3% to 45.9% in P24 and from 6.7% to 45.2% in P37 (Figure 2).

Although the following considerations are based on relative abundance data calculated only from the OTUs assigned at genus level (50% confidence cut-off), it is rather evident that the sea samples did not show a marked predominance of some taxa, as occurred in the ponds. In any case, the sea samples showed the highest diversity of major genera. *Pontimonas*, *Marivita*, *Escherichia*/*Shigella*, and *GpIIa* were constantly found with rather high abundances.

In P5, *Pontimonas* always notably dominated the bacterial communities, ranging from 18.7% to 61.7%. Except for *Lentibacter* and *Marivita* in two samples, all the other taxa were detected with Ras of about ≤10%.

In P24, *Pontimonas* showed broader abundance variations (Ra 8.4–85.8%). It dominated mainly in the period May 2013–April 2014, but in some months other genera predominated. For instance, *Thiohalocapsa* was always a rare taxon except in August 2012 when it showed a notable abundance of 35%. *Marivita* and *Bordetella* were particularly abundant from January to July 2013, and *Spiribacter* from May to November 2012. *GpVII* was present as major genus in same months, in particular in December 2012 (Ra = 26.4%).

In P37, except for *Thiohalocapsa*, all the taxa mentioned above increased their abundance and they were present as major genus in a higher number of samples. Moreover, *Salinibacter,* that in the other sampling sites was usually a rare taxon, was in this pond among the major taxa in various months, with abundances in the range 1.3–25.4%. By contrast, a strong reduction of *Pontimonas* was observed mainly in the first sampling year.

Unlike the samples collected from the sea, those taken from the ponds showed a constant presence of *Pontimonas* as major taxon, with very high abundance (mainly in P5 and P24) (Figure 2). However, this taxon seemed to be more abundant at low-intermediate salinities (20%–142‰) (Figure S5, Supplementary Materials).

### 2.2. Alpha- and Beta-Diversity of the Bacterial Communities

The α-diversity indices of the bacterial communities of ST samples collected from the sea and the selected ponds along the salinity gradient are reported in Table S2, Supplementary Materials. The number of OTUs ranged from 86 to 397, from 112 to 254, from 253 to 712 and from 129 to 455 for the sea, P5, P24 and P37, respectively. The Shannon index ranged from 1.871 to 4.660, from 1.480 to 3.857, from 3.518 to 5.843 and 2.565 to 4.578 for the sea, P5, P24 and P37, respectively. The Gini inequality index was very high in all sample ranging from 0.976 to 0.999.

Overall, P24 bacterial communities showed higher diversity (higher number of OTUs and Shannon Index) than those of sea, P5 and P37, and were the least dominated (lowest Gini Index)

The Canonical Correspondence Analysis (CCA) analysis showed that the community structure changed significantly with sampling site, sampling year and, salinity and showed seasonal variations, as accounted for by variables *sin*(Month), *cos*(Month) and their interaction with the sampling site (Table 1).

**Figure 3:**
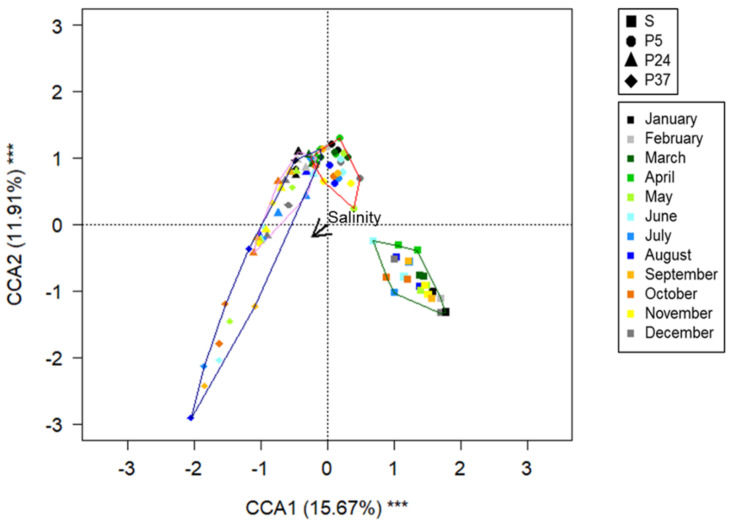
CCA ordination biplot showing samples according to the parameters driving the bacterial community structure. Arrow represents salinity. Variance explained by the two axes and axes significance (***p < 0.001) is reported. Line contour encloses samples collected from the same sampling site (green line: sea samples; red line: P5 samples; purple line: P24 samples; dark cyan line = P37 samples).

The first axis of CCA plot (Figure 3) accounted mainly for different sampling sites. Based on this parameter, samples collected from the ponds P37 and P24 formed overlapping clusters and were distributed along the salinity gradient, whereas bacterial communities of P5 and S clustered separately.

### 2.3. Variation of α-Diversity Indices and General Abundance in Relation to the Parameters Structuring the Bacterial Communities

Generalised Linear Models (GLMs) were run to analyse whether α-diversity indices and relative abundance of the most abundant genera significantly varied according to the parameters that structured the bacterial communities (see Table 1)

The α-diversity indices did not show significant variations (*p* > 0.05), whereas some taxa changed significantly according to the tested parameters.

**Figure 4:**
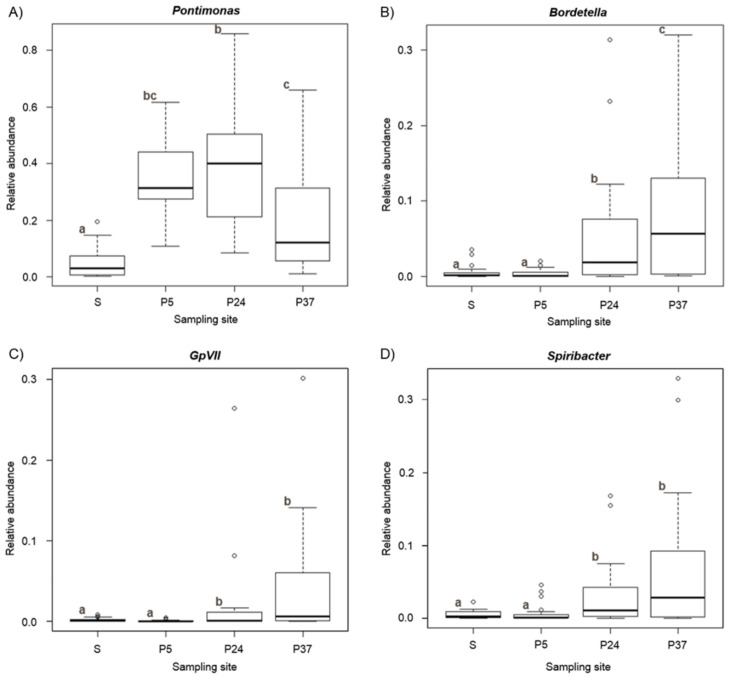
Box-whisker plot (Generalised Linear Model (GLM) analysis) showing abundance variation of *Pontimonas* (**A**), *Bordetella* (**B**), *GpVII* (**C**) and *Spiribacter* (**D**) across the sampling sites. The thick lines represent the median, boxes’ upper and lower limits indicate the 25th and the 75th percentiles respectively, whiskers indicate the data that go beyond the 5th percentile (lower whisker) and the 75th percentile (upper whisker), and dots represent the outliers. Different letters indicate differences between the mean values of different groups.

Among the most abundant genera (*Pontimonas*, *Marivita*, *Spiribacter*, *Bordetella*, *GpVII* and *Lentibacter*), *Lentibacter* and *Marivita* showed significant variations among the sampling sites (Figure 4). *Pontimonas* (F_3,79_ = 55.571, *p*_FDR_ < 0.001) showed the lowest relative abundances in the sea, increasing in P5 and P24, and decreasing again in P37; no statistically significant differences were recorded between the sea and P37 and between P5 and P24. *Spiribacter* (F_3,79_ = 14.767, *p*_FDR_ < 0.001) and *GpVII* (F_3,79_ = 31.110, *p*_FDR_ < 0.001) showed a similar trend among sampling sites, being lower in the sea and P5, and higher in P24 and P37 (with no statistically significant differences between the sea and P5 and between P24 and P37). *Bordetella* (F_3,79_ = 25.274, *p*_FDR_ < 0.001) was very low in the sea and P5 (with no statistically significant differences between these sites), increasing in P24 and even more in P37.

**Figure 5:**
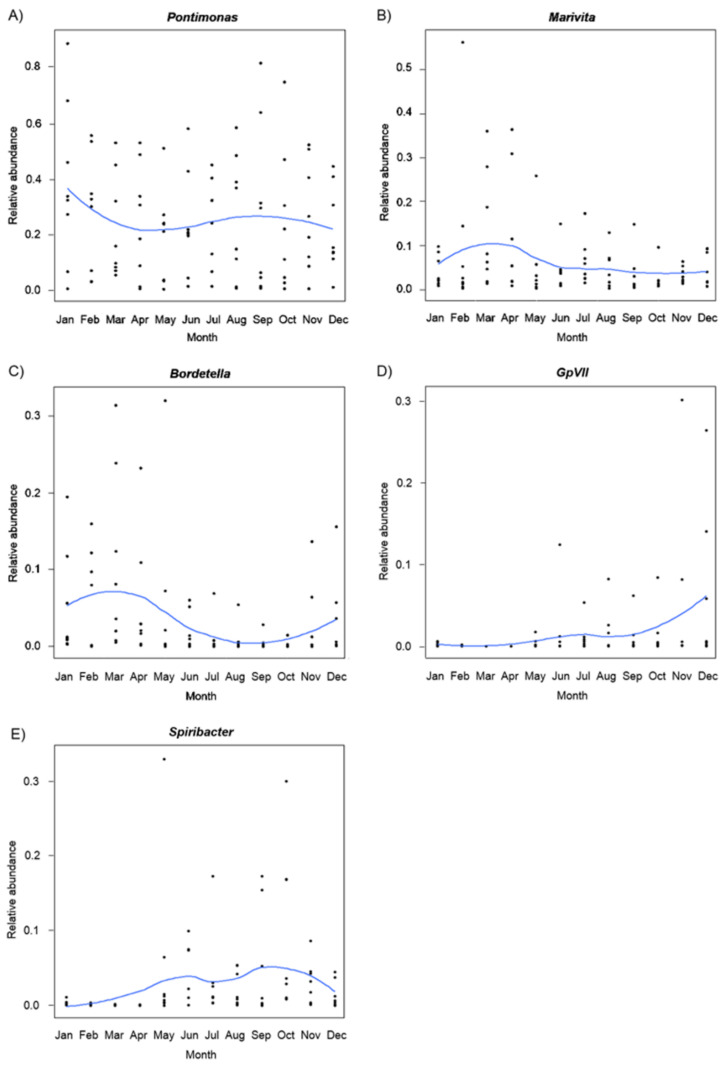
GLM plots showing seasonal variation in abundance of *Pontimonas* (**A**), *Marivita* (**B**), *Bordetella* (**C**), *GpVII* (**D**) and *Spiribacter* (**E**).

Taxa seasonal variation was also investigated (Figure 5). *Pontimonas* showed a maximum in January, then decreased reaching a minimum in spring, and increased slightly in autumn (*sin*(Month): F_1,79_ = 26.475, *p*_FDR_ < 0.001). *Marivita* had similar abundances from June to January (from late summer to winter) and reached a maximum in spring (*cos*(Month): F_1,79_ = 12.027, *p*_FDR_ = 0.006). *Spiribacter* showed a minimum in winter, increasing until June (early summer), then it experienced a slight decrease, only to rise again reaching a maximum in autumn (*sin*(Month): F_1,79_ = 16.174, *p*_FDR_ < 0.001; *sin*(Month) × *cos*(Month): F_1,79_ = 35.472, *p*_FDR_ < 0.001). *Bordetella* had a maximum in spring and a minimum in autumn (*sin*(Month): F_1,79_ = 9.478, *p*_FDR_ = 0.014). *GpVII* was usually quite low but increased from September to reach a maximum in December (it increased from the early autumn to reach a maximum in early winter) (*cos*(Month): F_1,79_ = 41.793, *p*_FDR_ < 0.001).

*Marivita* varied also with the interaction *sin*(Month) × sampling site (F_1,79_ = 8.7661, *p*_FDR_ < 0.001).

**Figure 6:**
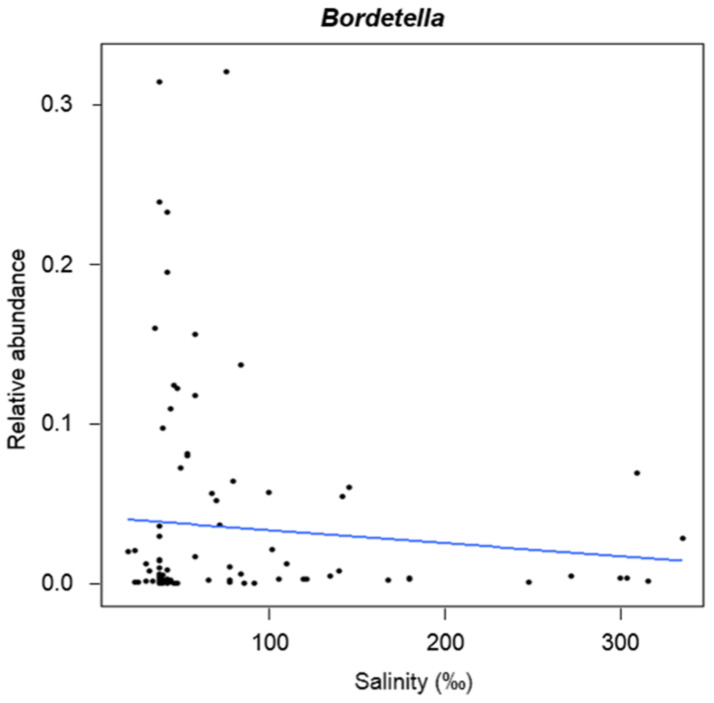
GLM plot showing variation in abundance of *Bordetella* in relation to the salinity.

Only *Bordetella* showed significant variation (z = 12.170, *p*_FDR_ < 0.001) in relation to salinity (Figure 6), decreasing with increasing salinity.

**Figure 7:**
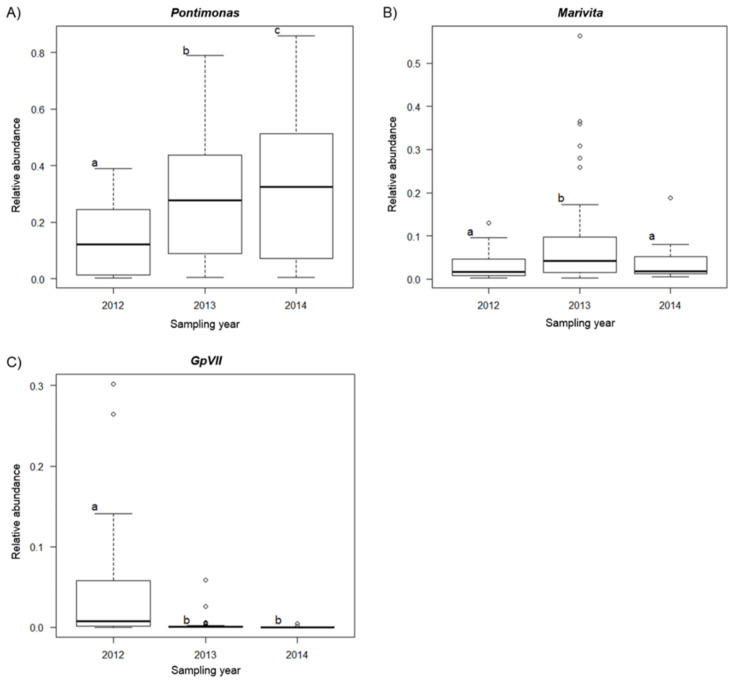
Box-whisker plot (GLM analysis) showing abundance variation of *Pontimonas* (**A**), *Marivita* (**B**) and *GpVII* (**C**) in relation to the sampling years. The thick lines represent the median, boxes’ upper and lower limits indicate the 25th and the 75th percentiles respectively, whiskers indicate the data that go beyond the 5th percentile (lower whisker) and the 75th percentile (upper whisker), and dots represent the outliers. Different letters indicate differences between the mean values of different groups.

*Pontimonas*, *Marivita* and *GpVII* varied according to the sampling year (Figure 7). *Pontimonas* abundance increased progressively from 2012 to 2014 (F_2,79_ = 26.876, *p*_FDR_ < 0.001). *Marivita* was higher in 2013 than in 2012 and 2014 (F_2,79_ = 24.3394, *p*_FDR_ < 0.001). *GpVII* was higher in 2012 than in 2013 and 2014 (F_2,79_ = 133.8325, *p*_FDR_ < 0.001).

## 3. Discussion

The current work was aimed at extending knowledge of hypersaline environments especially investigating bacterial communities’ variations in relation to the salinity gradient established along different ponds within a MS system (Saline di Tarquinia, Italy). The study considered the bacterial community assemblages starting from the neighbouring sea (representing the saltern seawater inlet) up to the crystallisation ponds.

Although some of the ST ponds have already been preliminarily studied by DGGE [7], the ST environment deserves more detailed investigation. This work represented the first in-depth characterisation of these bacterial communities by a high throughput metabarcoding approach. In addition, the value of the current investigation is broader, since the study was systematically planned, comprising a two-year survey with monthly sampling of various ponds.

Our outcomes will be compared to those obtained by other authors regarding the bacterial communities of water samples of various MS worldwide (see below).

This work evidenced that all the bacterial communities found along the ST salinity gradient were strongly uneven, as suggested by the high values (0.976–0.999) of the Gini index (Table S2, Supplementary Materials). The CCA analysis (Table 1, Figure 3) showed that the community variation was related to the sampling site, salinity, sampling year and month (addressing a seasonality effect). Based on the combination of these factors, the communities were clearly ordered according to the salinity gradient. As expected, the largest differences occurred between the communities of the sea and P37.

The taxonomic analysis revealed that these bacterial communities were dominated by members of three phyla: *Proteobacteria*. *Bacteroidetes* and *Actinobacteria*. In general, they are also retrieved as the most abundant bacterial taxa in other solar salterns around the world [17,18,20,24,28,30,31,33,36,52,53].

The progressive variation of the bacterial community assemblages along the salinity gradient (from the neighbouring sea towards the crystallisation ponds), which was evident at the genus level, was already revealed at a high taxonomic level (Phylum).

*Proteobacteria,* the most abundant phylum in the sea, decreased in P5 where a codominance of *Proteobacteria* and *Actinobacteria* was observed. The increase of *Actinobacteria* in P5 occurred concomitantly with a decrease of *Bacteroidetes*. This result was somehow surprising, the salinities recorded in the sea and P5 being quite similar; thus, a similar community composition was expected.

In P24, *Proteobacteria* were higher again in the first year, and with even more variations, its abundances very similar to those of the sea. By contrast, in the second year, relative abundance of this phylum was quite low and a preponderance of *Actinobacteria* occurred. In P24, relative abundance of *Bacteroidetes* was always lower than in P5, but it increased in P37 (crystallisation pond), particularly in months with higher salinity.

In any case, in all sites both *Proteobacteria* and *Actinobacteria* were higher at low-intermediate salinities (26%–180%). Similar results were reported by other authors [24,26,28,31,34,36,53]. However, a decrease in the relative abundance of these phyla along the salinity gradient was often reported and, at salinities higher than 200‰, *Bacteroidetes* abundance highly increased [4,19,22,28,30,31,34,36,37,54]. Moreover, various studies evidenced that at the highest salinities (≥ 300‰), where diversity is low, *Euryarchaeota* (Archaea) represented the greatest proportion of the community, but *Bacteroidetes* was the most abundant phylum among Bacteria (often representing almost the entire bacterial community) [4,17,28,29,31,34,37,53,54,55,56,57]. This was not confirmed by our work, since in ST *Bacteroidetes* generally showed low abundances, excluding some peaks in months characterised by high salinities. However, it was lower than *Proteobacteria* and *Actinobacteria,* which also dominated at high salinities. Thus, our outcome was somehow inconsistent with the majority of results from others, but it must be taken into account that most of these regarded the Santa Pola saltern (Spain), which is considered a study model for hypersaline environments, but is located in a more southerly location than ST. Actually, for different solar salterns over the world, other authors reported quite different assemblages concerning the bacterial fraction of the prokaryotic communities, indicating possible bio-geographic patterns [18,20,21,23,26,27,32,33,34,35,38,39]. Among them, some studies regarding the MS of Chile, Mexico, India, Bulgaria, and Spain revealed not only the presence of *Proteobacteria* in the crystalliser ponds (280%–440‰ of salinity), but also that they were more abundant than *Bacteroidetes* [23,27,33,35,38,39]. In addition, in some cases, *Actinobacteria* was also found among the major phyla harboured by the crystallization ponds [32,33,38].

The taxonomic analysis at the genus level evidenced a relatively high number of major taxa in the sea, which showed, in general, low-medium abundances. Instead, along the salinity gradient from the concentrator ponds to the crystallisation ponds, the taxonomic pattern (regarding major taxa) was simplified and an evident preponderance of some genera occurred.

An interesting outcome of this work regarded *Pontimonas*; it was relatively scarce (<20%) in the sea, but usually quite abundant in the ponds up to ca. 200‰ of salinity (Figure 2, Figure S5, Supplementary Materials). In addition, at the highest salinities of P37 it was always a major genus even if with much lower abundances (1.2–6.1%). This genus includes only one species (*P. salivibrio*) and the only cultivable strain (CL-TW6) was isolated in Korea from a solar saltern pond showing salinity slightly higher than seawater. It was assumed to be a slightly halophilic marine bacterium (optimal growth at 30‰ of salinity) by the researchers that described the species [58,59], who supposed that it was drawn into the saltern from the coastal environment [60]. No other work reports isolation of P*ontimonas* strains from MS or other hypersaline environments. Instead, some molecular studies also revealed the presence of this bacterium in continental hypersaline lakes at higher salinities [61,62]. In addition, to the best of our knowledge, the presence of *Pontimonas* in a MS was reported only by Leoni et al. (2020); this metabarcoding study found this genus at intermediate salinity (131% and 145‰), but with quite low relative abundance (~2%) [34].

In our case, *Pontimonas* relative abundances were lowest in the sea and highest mainly in P5 and P24 (although abundances in P37 were not statistically different from those in P5) (Figure 2, Figure 4A). Since salinity levels in the sea and P5 were quite similar, the very high differences in their *Pontimonas* abundance (Figure 2) could be due to other variables.

Due to its possible salinity preferences, this genus, even if with some exceptions, could be considered as a slight and/or moderate halophile. However, the constant presence of this genus in P37, and its rather high abundances (30–38%) at high salinities (Figure S5, Supplementary Materials), would also suggest a possible behaviour as a borderline-extreme halophile or, at least, as a moderate halophile with broad euryhalinism. Since these results were obtained over two years, they strongly reinforce the hypotheses of an evident adaptation of this genus to broad salinity variations, which could be substantiated also by the physiological data regarding the ST bacterial strains showing marked euryhalinism [3].

The presence of *Bordetella* in ST deserves to be discussed in detail. *Bordetella* species were mainly isolated from warm-blooded animals (including humans) and, until recently, they were thought to be only animal pathogens [63,64]. Nonetheless, recent studies revealed that *Bordetella* strains could also have an environmental origin [64,65,66]. It has been assumed that the *Bordetella* species related to animals evolved from ancestral environmental strains, and some animal-adapted species still maintain the ability to proliferate in the environment [64,67]. Among this genus, some members are known to be avian-associated microorganisms [68,69,70].

In our case, we have no definite information to explain the presence of this taxon, but since ST hosts a great avian biodiversity [71,72], we can assume that the presence of *Bordetella* in ST could have a primary avian origin with subsequent adaptation to the saltern environment, where it is quite diffused and not sporadic. In fact, this genus was constantly found in all samples; in the sea and P5 it often represented a rare taxon, but in P24 and P37 it was frequently a major genus with some high abundances (Figure 2). Moreover, GLM indicated a main seasonal occurrence of this taxon, which increased in spring and decreased in autumn (Figure 5C). It can be speculated that the total bacterial abundance in the high-salinity ponds can be lower than in low-salinity ponds. Therefore, exogenous bacteria brought by animals (e.g., birds) into these ponds might become major taxa even if they could be unable to grow.

The metabarcoding survey confirmed the presence of *Spiribacter* and *Salinibacter* in ST, like in other MS worldwide [23,26,27,28,31,36,52,54,73,74]. However, they have a different distribution with respect to salinity: *Spiribacter* generally dominates in the concentrator ponds with salinities of 100%–250‰, while *Salinibacter* dominates in the crystallisation ponds [19,28,31,34,74,75,76,77]. Presence of these genera at the mentioned specific conditions was confirmed also in our study, but they were not dominant. Absence or scarce abundance of *Salinibacter* in the crystalliser ponds have been pointed out also by other authors who studied MS of India, Spain and Greece [20,38,39].

However, as already stated by other authors, changes in environmental and or climatic conditions may influence saltern microbial communities; thus, in different geographic areas, different microbial assemblages could be revealed [19,27,37,54].

## 4. Materials and Methods

### 4.1. Sample Collection and Characterisation

**Figure 8:**
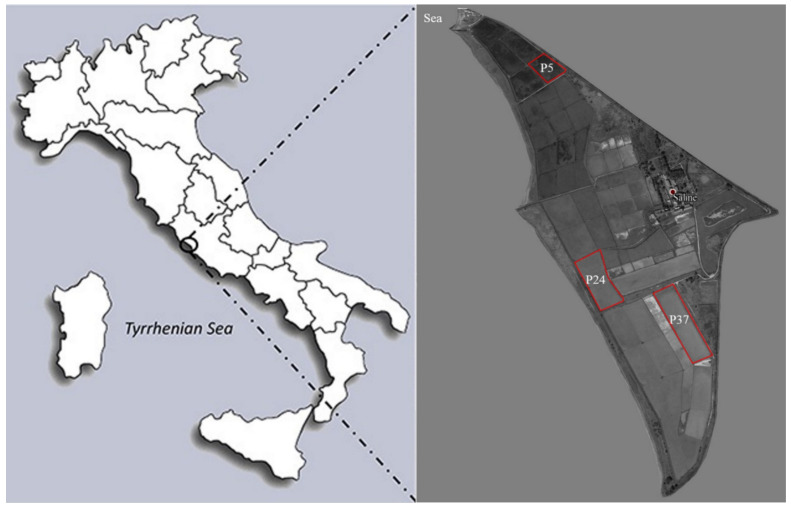
Detailed map of the “Saline di Tarquinia” (Viterbo, Italy) marine salterns with indication of the sampling sites (Sea, P5, P24 and P37). Map was generated using Google Earth Pro version 7.3.1 and graphically edited.

Water samples were collected monthly over two years (from May 2012 to April 2014) from three ponds (P5, P24 and P37) within the ST (North Tyrrhenian Sea, Italy; 42°12′07.8′′ N 11°43′17.8′′ E) and from the nearby sea (Figure 8). The three non-consecutive ponds (low-salinity concentrator pond, P5; intermediate-salinity concentrator pond, P24; crystallisation pond, P37) were selected according to their annual range of salinity in order to cover the whole ST gradient.

All samples, obtained by pooling three different sub-samples [1,39,78], were stored in sterile bottles and kept refrigerated (4 °C) during the transport to the ST laboratory to be processed within an hour. Then, 1 L of water was vacuum filtered on sterile membranes (0.22 µm, Millipore, Burlington, MA, USA), which were washed twice with sterile saline (NaCl) solutions (with concentration roughly corresponding to the salinity recorded on the sites) to remove possible nutrients from the filters [78]. Membranes were maintained frozen until DNA extraction. The remaining sample aliquots were used to determine BOD5 and chlorophyll pigment concentration, which were measured by standard methods [79]

Environmental parameters (salinity, pH, conductivity and water temperature) were recorded during sampling by common handheld probes (see Table S1, Supplementary Materials for details on the recorded parameters over the sampling period). Daily rainfall and average temperatures of the site were obtained from a local government institution (“Ufficio Idrografico e Mareografico, Centro Funzionale Regionale, Regione Lazio”; [80]).

### 4.2. DNA Extraction, 16S rDNA Amplicon Libraries and Sequencing

DNA was extracted from the membranes using a GeneMATRIX Bacterial & Yeast Genomic DNA Purification Kit (EURx Ltd., Gdańsk, Poland) as previously reported [81,82]. The multiplexed libraries were prepared using a dual PCR amplification protocol (targeting the V5–V6 hypervariable regions of 16S rRNA gene) as previously reported [83,84]. The first PCR was carried out in 3 × 80 μL volume reactions using the GoTaq Green Master Mix (Promega Corporation, Madison, WI, USA) and 1 μM of each primer. The primers used, 783F and 1046R [85,86], were modified by adding external barcodes to allow the parallel processing of multiple samples. The PCR cycling conditions were: 98 °C for 30 s; 20 cycles at 98 °C for 10 s, 47 °C for 30 s, and 72 °C for 5 s and a final extension at 72 °C for 2 min. The amplicons were purified by Wizard SV Gel and PCR Clean-up System (Promega Corporation, Madison, WI, USA) and quantified using Qubit 2.0 (Life Technologies, Carlsbad, CA, USA). Then, the purified amplicons were pooled to obtain a single library (each library contained nine samples, identifiable by different external barcode pairs). The second step of library preparation, with the addition of standard Nextera indexes (Illumina, Inc., San Diego, CA, USA), and sequencing was carried out at Nuova Genetica Italiana SRL (Monza-Brianza, Italy). Amplicon libraries were sequenced by Illumina MiSeq (Illumina Inc., San Diego, CA, USA) using a 2 × 250 bp paired-end protocol.

### 4.3. Sequence Processing and Data Analysis

Reads from sequencing were demultiplexed according to the indices and internal barcodes. Reads/sequences were processed using the UPARSE pipeline [87]. Forward and reverse reads were merged with perfect overlapping and quality filtered with default parameters. Suspected chimeras and singleton sequences (i.e., sequences appearing only once in the whole data set) were removed. OTUs were defined on the whole data set clustering the sequences at 97% of similarity and defining a representative sequence for each cluster. The abundance of each OTU was estimated by mapping the sequences of each sample against the representative sequence of each OTU at 97% of similarity. Taxonomic classification of the OTU representative sequences was obtained by RDP classifier [88], using 50% confidence cut-off as suggested for short sequences [89].

Sequence data were submitted to the European Nucleotide Archive (ENA), study accession number PRJEB38856 (http://www.ebi.ac.uk/ena/data/view/PRJEB38856).

### 4.4. Statistical Methods

The coverage of each sample was evaluated by the Good’s method. The percentage of coverage of each sample was estimated using the formula [1 − (n/N)] × 100, where *n* is the number of OTUs in a specimen represented by one clone (singletons) and N is the total number of sequences in that specimen [90].

The Shannon index and Gini index were used to describe alpha diversity. The Shannon index takes into account the uniformity of species and its abundance. It ranges from 0 to +∞ and increases as both the richness and the evenness of the community increase [91]. The Gini index [92], developed as a measure of inequality of income in economics, is used in ecology to measure community evenness. Its values range from 0 to 1 and increasing values indicate a decrease in evenness [93]. The Shannon and the Gini indices were calculated on samples rarefied to 1929 randomly chosen sequences, corresponding to the minimum number of sequences at a sample (Table S2, Supplementary Materials). Analyses of alpha diversity indices were performed using linear models after checking that no relevant deviation from model assumption had occurred by visual inspection of residual plots (details not shown).

Beta diversity analyses were run on the non-rarefied samples [94], the number of sequences as abundance estimation of each OTU in a sample. Canonical Correspondence Analysis (CCA) was used to investigate the environmental parameters driving the community structure. Salinity, chlorophyll concentration, sampling month and sampling year were used as predictors. Sampling month data were entered as Fourier series transformed data to account for seasonality [95]. Other variables such as water temperature, pH, rainfall and BOD5 were removed because they were collinear with the abovementioned predictors (Pearson|r| > 0.6). CCA significance was assessed through 9999 permutations.

The abundance variation of the most abundant genera (with at least 40,000 sequences in the whole dataset) in relation to the parameters driving bacterial communities’ structure was investigated by Generalized Linear Models (GLMs), assuming a Poisson distribution and corrected for overdispersion. The log of the total number of sequences at a sample was also entered in the model as an offset, so that the GLMs model the proportion of sequences of a genus [96]. In this case, to account for taxa seasonal variation the periodic regression based on Fourier series transformation of month was also used [95]. Post-hoc tests were used to assess pairwise differences in taxa abundance variation among sampling sites. In GLMs, we accounted for multiple statistical tests by correcting *p*-values according to the false discovery rate (FDR) procedure [97].

All the statistical analyses were performed in R environment (R 3.4.2, R Core Team, 2018) using VEGAN, BIODIVERSITYR, MULTTEST, and MULTCOMP packages.

## 5. Conclusions

As well as their archaeal community, marine saltern ecosystems harbour interesting communities of halophilic bacteria, which are often considered somehow less important due to their much lower abundance particularly at the highest salinities and are analysed together with the other prokaryotes. Moreover, various studies have been carried out to assess the prokaryotic diversity of these environments, but the majority used low-resolution methods (i.e., 16S rDNA clone libraries or DGGE) returning a limited vision of the diversity and a distorted community structure. In addition, scant information is available regarding taxa fluctuations over time, most of the investigations being based on a one-time sampling.

Furthermore, although general community distribution along the MS salinity gradients have been described, it has been shown that MS of different geographic areas revealed only partially similar patterns of bacteria. Therefore, tracing and identifying common and different features in the bacterial community assemblages of the MS worldwide is of valuable biological and ecological interest.

Thus, the current work was specifically aimed at obtaining an in-depth characterisation of the bacterial communities found along the salinity gradient within the “Saline di Tarquinia” marine salterns. A metabarcoding approach was used to obtain detailed information on their community structure and composition and their spatial (along the salinity gradient) and temporal (over two years) variations.

In particular, the study evidenced that the ST communities showed, along the salinity gradient, an increasing simplification of bacterial diversity (particularly if compared to those of the nearby sea) and an increase of a few dominant taxa. Moreover, the variation of the bacterial communities along the salinity gradient was related to various factors: sampling site, sampling year, salinity, and sampling month.

The comparison with other marine salterns worldwide revealed different distribution of some bacterial groups, indicating a possible bio-geographic pattern.

Another interesting feature of the ST salterns was the constant presence of *Pontimonas* as major taxon along the whole salinity gradient (up to saturation) and with high abundance, mainly in the low-concentrator and intermediate-concentrator ponds. Until now, it has been described as a slightly halophilic marine bacterium and found only in two marine salterns, in Korea and South Italy. These findings could help to reconsider the adaptation characteristics of *Pontimonas* with respect to salinity.

The information obtained by the current study represents a valuable contribution to extend knowledge on the halophilic bacterial diversity of marine salterns, in particular on the composition and structure of the communities and their variations over time.

## Figures and Tables

**Table 1:** Canonical Correspondence Analysis (CCA) of variation of the operational taxonomic units (outs) abundance for sea, P5, P24 and P37 according to the parameters.

Variable	df	Variance	F	*p*
*sin*(Month) × cos(Month)	1	0.126	2.1049	0.001
Year	2	0.313	2.6093	0.001
Salinity	1	0.098	1.6318	0.027
Chlorophyll pigments	1	0.0711	1.1830	0.255
*sin*(Month) × Sampling site	3	0.3489	1.936	0.001
cos(Month) × Sampling site	3	0.3479	1.9209	0.001
Residuals	79	4.745		
F_10,79_ = 3.254, *p* = 0.001, adjusted R^2^ = 0.275				

## Data Availability

Sequence data are available at the European Nucleotide Archive (ENA), study accession number PRJEB38856 (http://www.ebi.ac.uk/ena/data/view/PRJEB38856).

## Supplementary Materials

The following are available online. Table S1. Environmental parameters recorded for the ST samples from May 2012 to April 2014, Table S2. Alpha-diversity indices of the ST amplicon libraries (Sea, P5, P24 and P37 samples), Figure S1. Box-whisker plot (GLM analysis) showing abundance variation of *Proteobacteria* (A), *Actinobacteria* (B), *Bacteroidetes* (C), *Cyanobacteria* (D) and *Firmicutes* (E) across the sampling sites. The thick lines represent the median, boxes upper and lower limits indicate the 25th and the 75th percentiles respectively, whiskers indicate the data that go beyond the 5th percentile (lower whisker) and the 75th percentile (upper whisker), and dots represent the outliers. The different letters indicate differences between the mean values of different groups, Figure S2. *Proteobacteria* distribution in the sampling sites according to the increasing salinities recorded in each site over the two-year survey, Figure S3. *Actinobacteria* distribution in the sampling sites according to the increasing salinities recorded in each site over the two-year survey, Figure S4. *Bacteroidetes* distribution in the sampling sites according to the increasing salinities recorded in each site over the two-year survey, Figure S5. *Pontimonas* distribution in the sampling sites according to the increasing salinities recorded in each site over the two-year survey.

## Sample Availability

Sample of the compounds are not available from the authors.
