# Supplementary material for: Spatio-Temporal Variation of the Bacterial Communities along a Salinity Gradient within a Thalassohaline Environment (Saline di Tarquinia Salterns, Italy)
Source: Molecules. 2021 Mar 2;26(5):1338. doi: 10.3390/molecules26051338 (PMC7958962; doi:10.3390/molecules26051338)
Supplement: Supplementary file 1 [file molecules-26-01338-s001.pdf]

Supplementary Material

Spatio-temporal Variation of the Bacterial Communities Along a Salinity Gradient within a Thalassohaline Environment (Saline di Tarquinia salterns, Italy)

Table S1. Environmental parameters recorded for the ST samples from May 2012 to April 2014.

Sample	Salinity (‰)	Water Temperature (°C)	pH	Conductivity (ms/cm)	Chlorophylls (µg/l)	BOD5 (mg/l)
May12-S	38	20.7	8.16	56.10	0.49	2.70
May12-P5	42	18.6	8.16	59.30	0.68	4.30
May12-P24	102	18.5	7.84	129.90	18.36	3.20
May12-P37	180	22.0	7.69	193.60	35.70	4.30
Jun12-S	38	25.8	8.28	57.77	0.95	3.80
Jun12-P5	42	27.2	8.31	61.73	0.57	2.70
Jun12-P24	122	32.6	7.50	140.93	46.99	7.60
Jun12-P37	304	36.1	7.20	299.00	30.57	6.50
Jul12-S	38	23.3	8.12	56.60	0.27	3.20
Jul12-P5	42	24.1	8.65	58.90	1.37	2.70
Jul12-P24	140	27.7	8.05	123.20	24.18	5.40
Jul12-P37	310	31.3	7.89	200.00	170.64	6.50
Aug12-S	38	27.3	8.10	57.77	1.18	2.10
Aug12-P5	46	25.5	8.50	65.69	0.30	2.70
Aug12-P24	142	25.5	8.15	121.13	54.75	5.40
Aug12-P37	316	29.3	7.60	332.99	10.95	3.80
Sep12-S	38	22.3	8.15	57.77	2.13	0.50
Sep12-P5	40	22.5	8.37	59.75	3.35	1.00
Sep12-P24	135	23.0	7.86	158.75	16.48	7.60
Sep12-P37	272	27.8	7.72	289.43	15.51	3.80
Oct12-S	38	20.9	7.90	57.77	0.76	0.50
Oct12-P5	40	20.6	8.30	59.75	0.84	0.50
Oct12-P24	120	24.1	7.90	162.71	21.67	2.70
Oct12-P37	180	25.8	7.90	198.35	28.06	2.70
Nov12-S	40	16.0	8.10	59.75	1.06	3.20
Nov12-P5	42	13.3	8.30	61.73	1.52	3.20
Nov12-P24	80	15.7	8.15	99.35	39.35	6.50
Nov12-P37	84	17.7	8.44	103.31	28.17	4.90
Dec12-S	38	17.0	8.15	57.77	0.38	1.60
Dec12-P5	30	17.1	8.17	49.85	3.80	1.60
Dec12-P24	72	17.5	8.08	91.43	30.23	5.40

Dec12-P37	58	17.8	8.20	77.57	15.17	3.80
Jan13-S	38	10.1	8.20	57.77	1.25	10.40
Jan13-P5	30	9.3	8.10	49.85	1.90	1.00
Jan13-P24	58	10.5	8.10	77.57	14.14	2.70
Jan13-P37	42	9.7	8.20	61.73	7.32	2.70
Feb13-S	38	11.8	8.15	57.77	0.11	0.50
Feb13-P5	24	9.4	8.26	43.91	0.15	1.00
Feb13-P24	48	12.2	8.00	67.67	0.23	1.60
Feb13-P37	35	11.5	8.14	54.80	0.38	2.70
Mar13-S	38	14.2	8.10	57.77	3.19	1.00
Mar13-P5	20	14.5	8.50	39.95	6.54	3.80
Mar13-P24	38	17.7	8.30	57.77	56.08	3.20
Mar13-P37	38	17.6	8.50	57.77	16.20	2.70
Apr13-S	38	15.0	8.22	57.77	2.62	7.10
Apr13-P5	24	14.7	8.05	43.91	1.37	2.70
Apr13-P24	42	16.5	8.36	61.73	5.93	6.50
Apr13-P37	44	17.3	8.17	63.71	12.32	2.20
May13-S	38	18.1	8.20	57.77	2.81	0.50
May13-P5	26	20.5	8.50	45.89	2.28	3.20
May13-P24	50	22.2	7.50	69.65	71.86	12.00
May13-P37	76	22.6	7.40	95.39	88.21	18.60
Jun13-S	38	27.3	8.10	57.77	1.56	0.50
Jun13-P5	38	27.5	8.10	57.77	2.36	2.10
Jun13-P24	70	28.4	7.50	89.45	20.76	7.60
Jun13-P37	146	30.1	7.50	164.69	18.48	6.50
Jul13-S	38	29.0	8.20	57.77	1.43	1.60
Jul13-P5	42	28.1	8.20	61.73	7.19	43.20
Jul13-P24	78	33.2	7.70	97.37	67.87	13.70
Jul13-P37	248	37.3	7.40	265.67	3.65	2.70
Aug13-S	38	27.4	8.16	57.77	0.59	6.00
Aug13-P5	44	27.9	8.81	63.71	16.96	2.70
Aug13-P24	78	31.1	7.99	97.37	45.34	25.70
Aug13-P37	300	34.7	7.90	188.45	39.35	26.30
Sep13-S	38	21.7	8.20	57.77	0.27	1.00
Sep13-P5	48	23.9	8.80	67.67	4.56	3.80
Sep13-P24	106	24.8	7.90	125.09	28.33	3.80
Sep13-P37	336	25.4	7.20	352.79	4.56	2.10
Oct13-S	38	23.5	8.20	57.77	0.91	2.10
Oct13-P5	42	24.0	8.50	61.73	8.14	3.20
Oct13-P24	92	24.3	8.00	111.23	22.58	7.10
Oct13-P37	168	25.9	8.10	186.47	27.88	6.00
Nov13-S	40	18.5	8.20	59.75	2.66	1.60
Nov13-P5	44	18.2	8.20	63.71	1.37	1.00
Nov13-P24	86	17.2	8.00	105.29	12.78	4.90

Nov13-P37	110	17.6	8.00	129.05	17.34	5.40
Dec13-S	40	16.9	8.30	59.75	0.87	1.60
Dec13-P5	42	13.9	7.90	61.73	4.11	2.70
Dec13-P24	84	17.0	8.00	103.31	7.98	4.20
Dec13-P37	100	15.1	8.10	119.15	19.39	2.10
Jan14-S	38	11.5	8.10	57.77	1.21	1.60
Jan14-P5	42	7.5	7.90	61.73	2.28	3.20
Jan14-P24	78	6.8	8.00	97.37	12.03	3.80
Jan14-P37	68	8.7	8.20	87.47	26.09	4.90
Feb14-S	38	11.6	8.10	57.77	0.46	1.00
Feb14-P5	40	10.3	8.10	59.75	0.68	2.00
Feb14-P24	54	12.6	8.20	73.61	15.72	7.10
Feb14-P37	40	12.6	8.20	59.75	6.92	4.90
Mar14-S	40	15.3	8.00	59.75	1.03	1.00
Mar14-P5	32	17.0	8.20	51.83	0.99	2.10
Mar14-P24	54	16.3	8.30	73.61	2.81	3.00
Mar14-P37	46	18.6	8.40	65.69	12.66	4.00
Apr14-S	38	17.2	8.10	57.77	1.37	4.30
Apr14-P5	34	18.6	8.50	53.81	1.06	6.50
Apr14-P24	58	19.6	7.90	77.57	7.19	3.00
Apr14-P37	66	21.5	7.90	85.49	2.51	3.00

Table S2. Alpha-diversity indices of the ST amplicon libraries (Sea, P5, P24 and P37 samples).

Sample	Number of sequences	Number of OTUs	Shannon Index	Gini Index
May12-S	24,920	311	4.512	0.994
Jun12-S	3550	180	2.796	0.998
Jul12-S	6384	126	2.526	0.999
Aug12-S	47,020	208	3.479	0.997
Sep12-S	101,769	215	3.605	0.997
Oct12-S	11,151	121	2.300	0.999
Nov12-S	28,577	164	2.965	0.998
Dec12-S	41,800	171	3.469	0.998
Jan13-S	31,410	113	2.721	0.999
Feb13-S	16,944	122	3.098	0.999
Mar13-S	5645	148	2.992	0.998
Apr13-S	7699	190	3.066	0.998
May13-S	87,407	124	3.261	0.998
Jun13-S	7467	162	3.258	0.998
Jul13-S	36,416	167	2.944	0.998
Aug13-S	2071	163	3.505	0.998
Sep13-S	40,563	138	2.816	0.999

Oct13-S	1929	144	3.205	0.998
Nov13-S	3148	188	3.255	0.998
Dec13-S	6346	196	3.830	0.997
Jan14-S	6813	86	1.871	0.999
Feb14-S	26,883	187	3.344	0.998
Mar14-S	9769	154	3.374	0.998
Apr14-S	71,956	397	4.660	0.991
May12-P5	4702	144	2.037	0.999
Jun12-P5	37,626	139	3.032	0.999
Jul12-P5	8906	142	2.556	0.999
Aug12-P5	36,128	156	3.018	0.998
Sep12-P5	83,433	142	2.686	0.999
Oct12-P5	29,773	112	1.480	0.999
Nov12-P5	18,217	200	3.164	0.998
Dec12-P5	98,851	254	3.697	0.996
Jan13-P5	10,317	189	3.166	0.998
Feb13-P5	18,968	164	3.591	0.998
Mar13-P5	8363	150	2.836	0.998
Apr13-P5	17,932	165	3.198	0.998
May13-P5	83,794	139	2.315	0.999
Jun13-P5	12,446	116	2.824	0.999
Jul13-P5	6199	141	2.736	0.999
Aug13-P5	3002	205	3.149	0.998
Sep13-P5	34,208	178	3.182	0.998
Oct13-P5	20,186	125	2.704	0.999
Nov13-P5	2593	150	3.123	0.998
Dec13-P5	21,027	230	3.857	0.997
Jan14-P5	3274	130	2.931	0.999
Feb14-P5	51,927	220	3.365	0.997
Mar14-P5	9742	251	3.281	0.997
Apr14-P5	46,388	198	3.445	0.998
May12-P24	12,419	378	4.405	0.992
Jun12-P24	8829	511	5.096	0.987
Jul12-P24	21,271	432	4.884	0.990
Aug12-P24	43,518	432	4.787	0.990
Sep12-P24	104,020	354	4.670	0.993
Oct12-P24	13,811	672	5.791	0.978
Nov12-P24	20,571	648	5.751	0.978
Dec12-P24	35,432	593	5.602	0.982
Jan13-P24	13,339	712	5.843	0.976
Feb13-P24	5766	395	4.690	0.992
Mar13-P24	5634	307	3.518	0.995
Apr13-P24	16,140	417	4.909	0.990
May13-P24	17,620	386	4.865	0.991

Jun13-P24	21,113	253	4.037	0.996
Jul13-P24	9588	313	4.511	0.994
Aug13-P24	8062	503	4.952	0.988
Sep13-P24	15,770	332	4.499	0.994
Oct13-P24	18,988	660	5.775	0.979
Nov13-P24	44,585	426	4.951	0.990
Dec13-P24	99,581	356	4.541	0.993
Jan14-P24	6236	402	4.528	0.992
Feb14-P24	48,891	706	5.809	0.977
Mar14-P24	18,477	314	4.605	0.993
Apr14-P24	19,010	462	4.972	0.989
May12-P37	36,961	171	2.684	0.998
Jun12-P37	25,182	185	3.001	0.998
Jul12-P37	8699	238	3.314	0.997
Aug12-P37	64,570	372	4.376	0.992
Sep12-P37	19,756	260	3.592	0.997
Oct12-P37	19,758	150	2.608	0.999
Nov12-P37	37,803	305	3.747	0.995
Dec12-P37	23,878	455	4.525	0.990
Jan13-P37	14,731	174	2.949	0.998
Feb13-P37	16,738	272	3.933	0.996
Mar13-P37	16,949	152	2.938	0.998
Apr13-P37	11,598	246	3.738	0.996
May13-P37	8222	276	3.463	0.996
Jun13-P37	19,303	295	3.358	0.996
Jul13-P37	22,545	231	3.537	0.997
Aug13-P37	20,578	393	3.931	0.993
Sep13-P37	16,783	129	3.013	0.999
Oct13-P37	56,533	192	3.190	0.998
Nov13-P37	12,300	248	3.441	0.997
Dec13-P37	2143	306	3.801	0.995
Jan14-P37	10,892	168	2.565	0.998
Feb14-P37	59,262	450	4.578	0.991
Mar14-P37	46,012	244	3.344	0.997
Apr14-P37	43,769	375	4.190	0.993

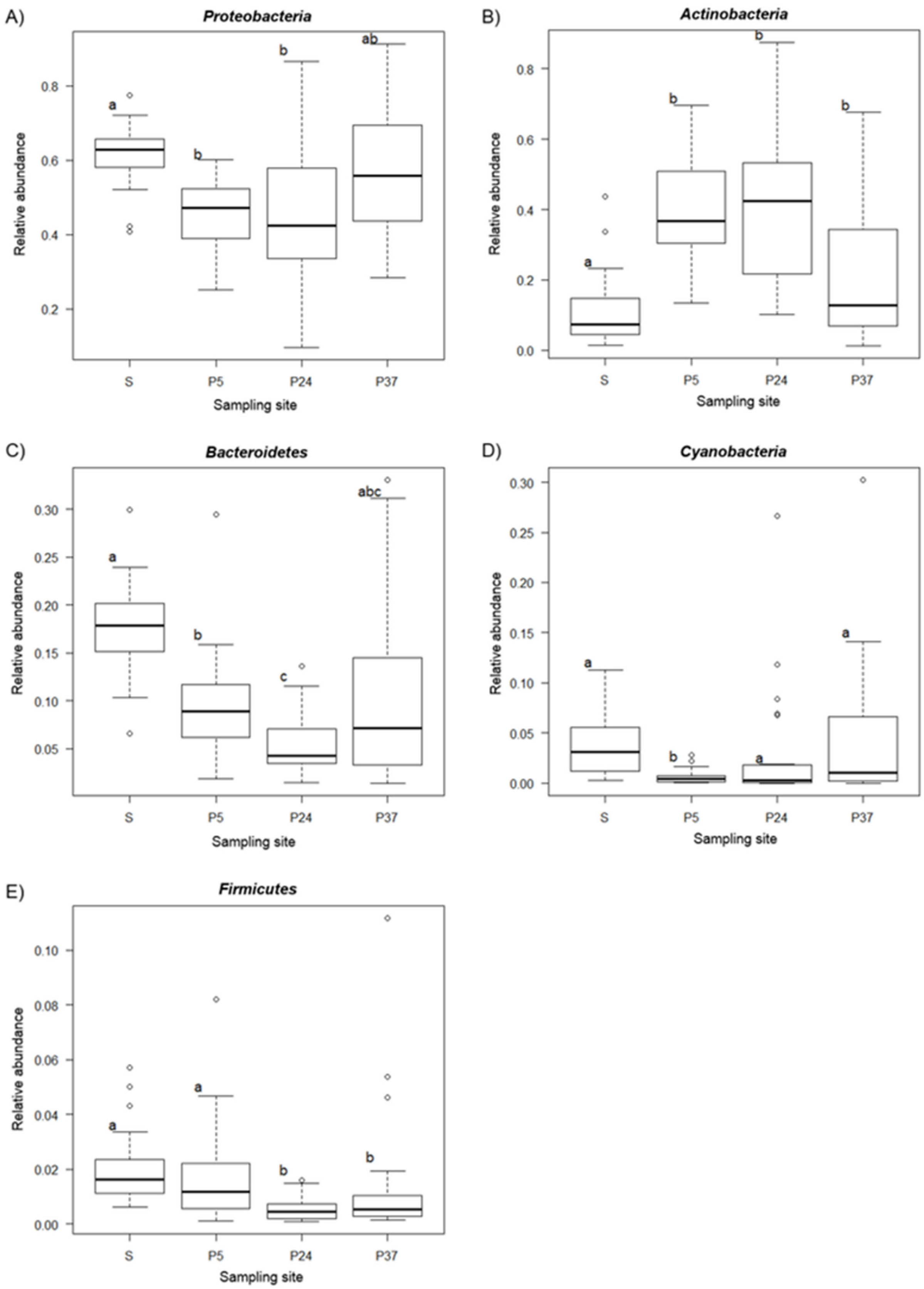

Figure S1. Box-whisker plot (GLM analysis) showing abundance variation of *Proteobacteria* (A), *Actinobacteria* (B), *Bacteroidetes* (C), *Cyanobacteria* (D) and *Firmicutes* (E) across the sampling sites. The

thick lines represent the median, boxes upper and lower limits indicate the 25th and the 75th percentiles respectively, whiskers indicate the data that go beyond the 5th percentile (lower whisker) and the 75th percentile (upper whisker), and dots represent the outliers. The different letters indicate differences between the mean values of different groups.

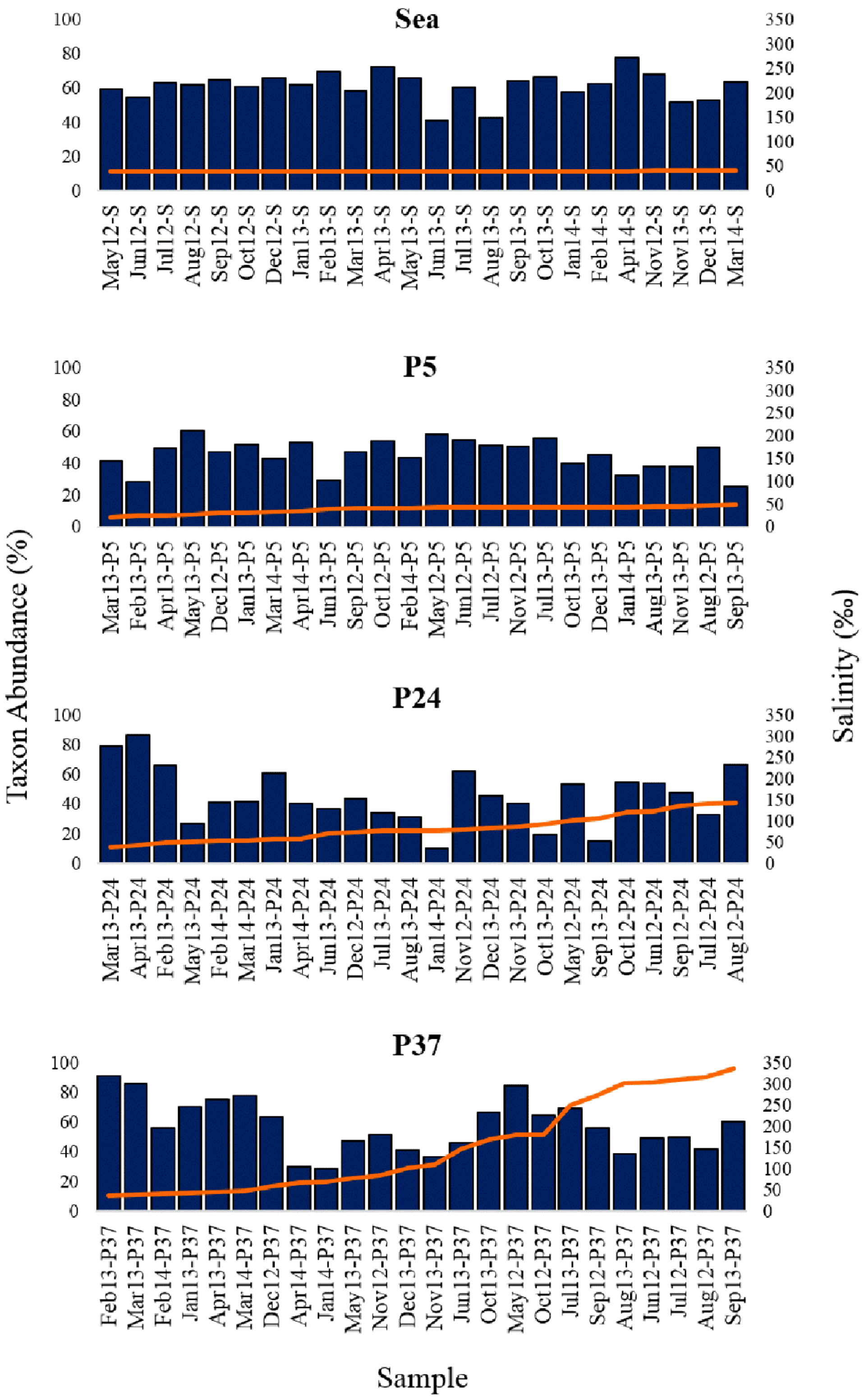

Figure S2. *Proteobacteria* distribution in the sampling sites according to the increasing salinities recorded in each site over the two-year survey

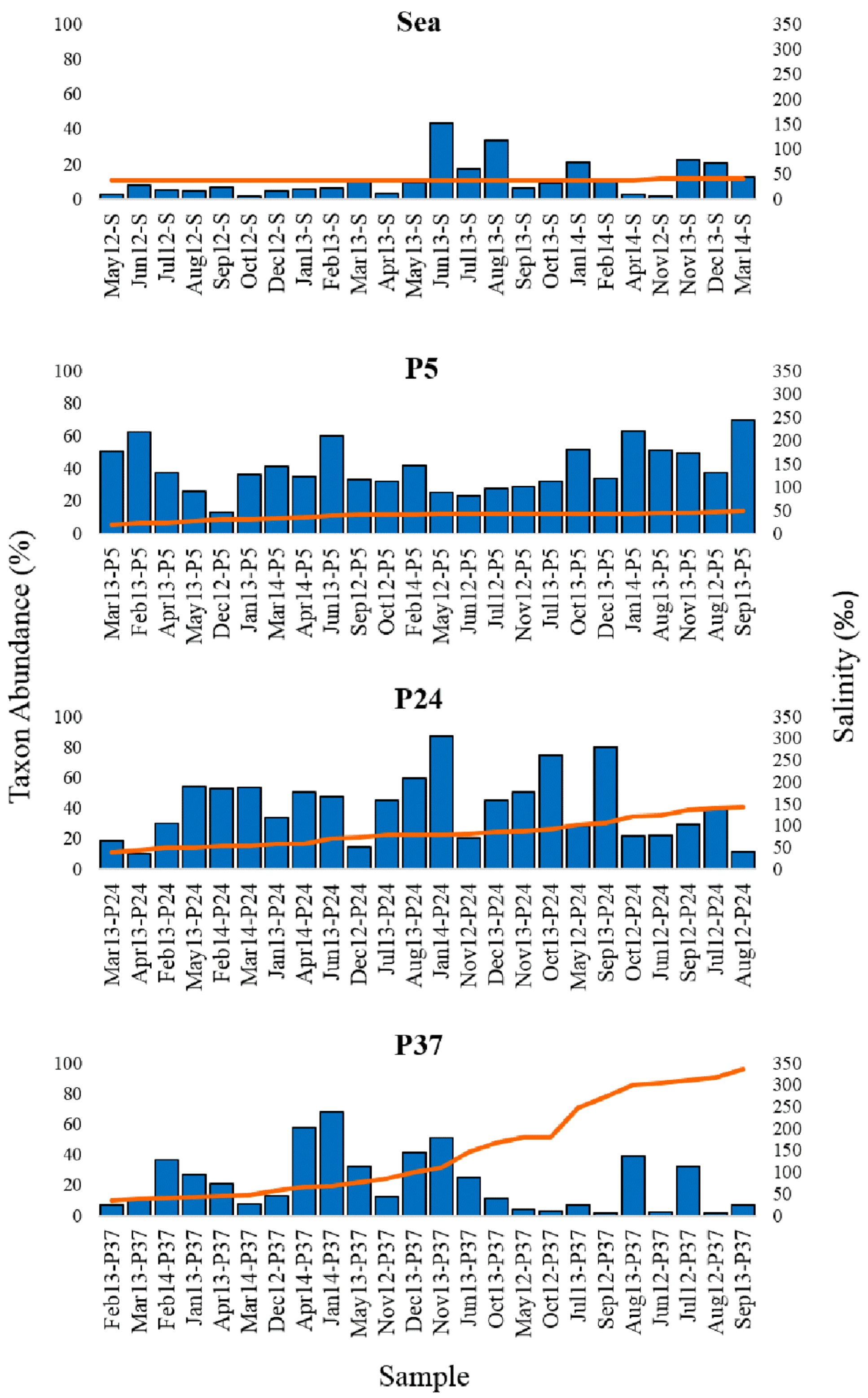

Figure S3. *Actinobacteria* distribution in the sampling sites according to the increasing salinities recorded in each site over the two-year survey

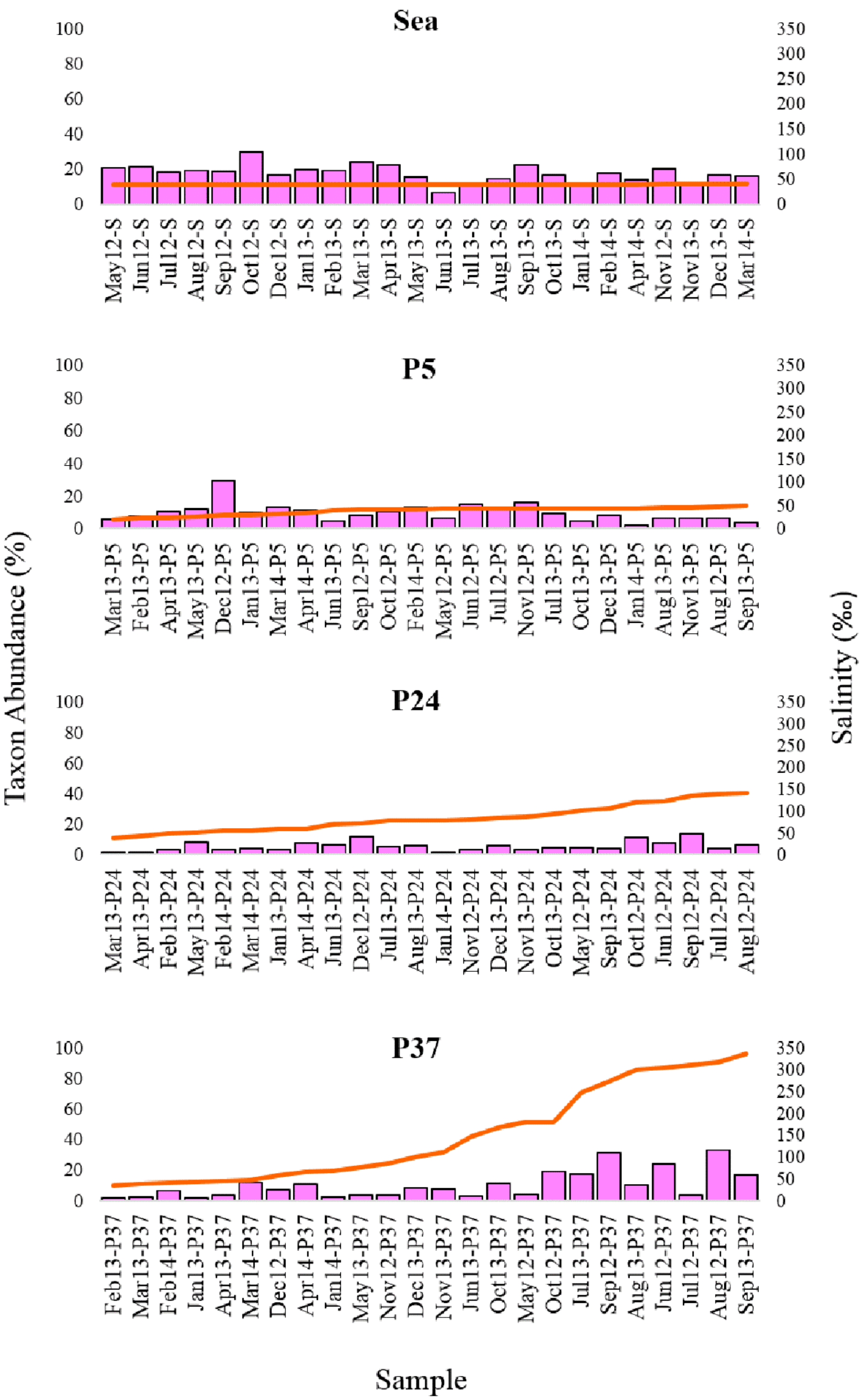

Figure S4. *Bacteroidetes* distribution in the sampling sites according to the increasing salinities recorded in each site over the two-year survey

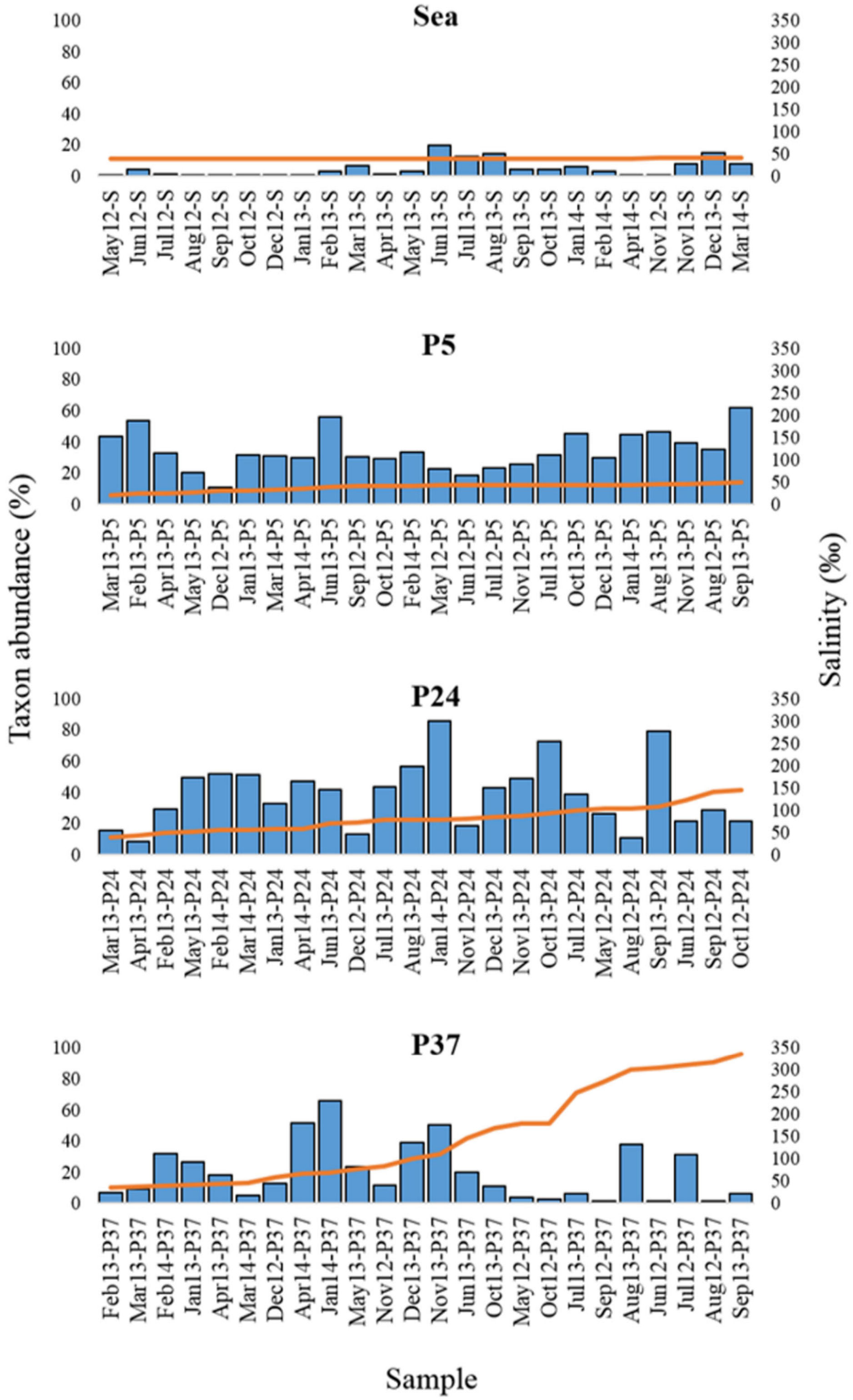

Figure S5. *Pontimonas* distribution in the sampling sites according to the increasing salinities recorded in each site over the two-year survey
